# miR-146a in Myasthenia Gravis Thymus Bridges Innate Immunity With Autoimmunity and Is Linked to Therapeutic Effects of Corticosteroids

**DOI:** 10.3389/fimmu.2020.00142

**Published:** 2020-03-10

**Authors:** Federica Bortone, Letizia Scandiffio, Stefania Marcuzzo, Silvia Bonanno, Rita Frangiamore, Teresio Motta, Carlo Antozzi, Renato Mantegazza, Paola Cavalcante, Pia Bernasconi

**Affiliations:** ^1^Neurology IV—Neuroimmunology and Neuromuscular Diseases Unit, Fondazione I.R.C.C.S. Istituto Neurologico Carlo Besta, Milan, Italy; ^2^Department of Pathological Anatomy, ASST—Bergamo Est Ospedale Bolognini Seriate, Bergamo, Italy

**Keywords:** myasthenia gravis, thymus, autoimmunity, innate immunity, miR-146a

## Abstract

Toll-like receptor (TLR)-mediated innate immune responses are critically involved in the pathogenesis of myasthenia gravis (MG), an autoimmune disorder affecting neuromuscular junction mainly mediated by antiacetylcholine receptor antibodies. Considerable evidence indicate that uncontrolled TLR activation and chronic inflammation significantly contribute to hyperplastic changes and germinal center (GC) formation in the MG thymus, ultimately leading to autoantibody production and autoimmunity. miR-146a is a key modulator of innate immunity, whose dysregulation has been associated with autoimmune diseases. It acts as inhibitor of TLR pathways, mainly by targeting the nuclear factor kappa B (NF-κB) signaling transducers, interleukin 1 receptor associated kinase 1 (IRAK1) and tumor necrosis factor (TNF) receptor associated factor 6 (TRAF6); miR-146a is also able to target c-REL, inducible T-cell costimulator (ICOS), and Fas cell surface death receptor (FAS), known to regulate B-cell function and GC response. Herein, we investigated the miR-146a contribution to the intrathymic MG pathogenesis. By real-time PCR, we found that miR-146a expression was significantly downregulated in hyperplastic MG compared to control thymuses; contrariwise, IRAK1, TRAF6, c-REL, and ICOS messenger RNA (mRNA) levels were upregulated and negatively correlated with miR-146a levels. Microdissection experiments revealed that miR-146a deficiency in hyperplastic MG thymuses was not due to GCs, but restricted to the GC-surrounding medulla, characterized by IRAK1 overexpression. We also showed higher c-REL and ICOS mRNA levels, and lower FAS mRNA levels, in GCs than in the remaining medulla, according to the contribution of these molecules in GC formation. By double immunofluorescence, an increased proportion of IRAK1-expressing dendritic cells and macrophages was found in hyperplastic MG compared to control thymuses, along with GC immunoreactivity for c-REL. Interestingly, in corticosteroid-treated MG patients intrathymic miR-146a and mRNA target levels were comparable to those of controls, suggesting that immunosuppressive therapy may restore the microRNA (miRNA) levels. Indeed, an effect of prednisone on miR-146a expression was demonstrated *in vitro* on peripheral blood cells. Serum miR-146a levels were lower in MG patients compared to controls, indicating dysregulation of the circulating miRNA. Our overall findings strongly suggest that defective miR-146a expression could contribute to persistent TLR activation, lack of inflammation resolution, and hyperplastic changes in MG thymuses, thus linking TLR-mediated innate immunity to B-cell-mediated autoimmunity. Furthermore, they unraveled a new mechanism of action of corticosteroids in inducing control of autoimmunity in MG via miR-146a.

## Introduction

Myasthenia gravis (MG) is a prototypical B-cell-mediated autoimmune disorder affecting neuromuscular junction, mainly caused by autoantibodies against the postsynaptic acetylcholine receptor (AChR), which lead to invalidating weakness and fatigability of skeletal muscles (1). The bulk of MG therapy consists of symptomatic treatment by acetylcholinesterase inhibitors, non-specific immunosuppression with corticosteroids, and thymectomy as a natural course disease-modifying intervention in selected patients (1–3). However, complete stable remission is only rarely achieved, and ~10% of patients are treatment refractory (2), highlighting the need to better understand the specific disease-associated pathogenic events, to develop more effective therapeutic strategies.

The involvement of thymus in AChR-MG pathogenesis is now widely accepted. This organ is the prime site of autosensitization and autoimmunity to AChR (4, 5), and thymectomy has beneficial effect in a high proportion of patients (6, 7). Approximately 80% of all AChR-MG patients presents thymic morphological and functional changes, including hyperplasia and thymoma (5). Hyperplasia is the most common alteration in early-onset MG; it is characterized by the expansion of perivascular spaces fused with the thymic medulla, which abnormally contains abundant B-lymphocyte infiltrates organized in ectopic germinal centers (GCs) forming follicles (8). In terms of immunoglobulin gene diversification, mutation, and selection, GCs in MG thymus do not differ from those observed in the lymphoid follicles of peripheral lymphatic organs, but they are uniquely surrounded by plasma cells and muscle-like myoid cells expressing AChR and other muscle proteins, which can set up an antigen-driven reaction (8). Chronic inflammation and persistent activation of Toll-like receptor (TLR)-mediated innate immune pathways have been critically implicated in the intrathymic MG pathogenesis, supporting the existence of a dangerous cross-talk between innate immunity and autoimmunity (9–13). Indeed, a hallmark of hyperplastic MG thymus is the significant overexpression of pro-inflammatory cytokines and chemokines [e.g. interleukin-6 (IL-6), type I interferons (IFNs), CXCL13, CCL21], along with upregulation of TLRs, particularly TLR3, TLR4, TLR7, and TLR9 (8, 10–13). The contribution of TLRs in autoimmunity can be explained by the ability of these receptors to stimulate maturation of antigen-presenting cells and production of type I IFNs and other inflammatory cytokines, which in turn cause priming of adaptive immune cells, such as autoreactive T cells (8). In addition, TLR7 and 9 stimuli may function as costimulatory signals for proliferation, maturation, and survival of B cells, thus compromising B-cell tolerance and promoting autoimmune response perpetuation (11, 12). Recently, Robinet and colleagues demonstrated that the combined use of TLR agonists induces thymic hyperplastic changes and triggers MG symptoms in mice, suggesting that tertiary lymphoid genesis, and consequently autoreactivity, in MG thymus could result from dysregulated TLR signaling (14). What exactly causes uncontrolled TLR activation, and loss of the fine regulation of TLR pathways, in hyperplastic MG thymus is not totally understood.

MicroRNAs (miRNAs) are ~22 nucleotide long small non-coding RNA molecules recognized to play a critical role in fine-tune regulation of gene expression (15). They modulate many biological processes, including cell-cycle progression, apoptosis, inflammation, and both innate and adaptive immune response (15). Thus, their involvement in several pathophysiological conditions, including cancer and autoimmunity (16–19), is not surprising. One of the most important miRNAs known to orchestrate immune and inflammatory signaling, and to play a central role in innate immunity, is miR-146a-5p (hereinafter called miR-146a) (20). Its dysregulated expression has been reported in different inflammatory and autoimmune pathologies, including systemic lupus erythematosus (SLE) (21), rheumatoid arthritis (RA) (22, 23), multiple sclerosis (MS) (24), and sepsis (25, 26). miR-146a gene is located within the MIR3142HG host gene on chromosome 5 (5q33.3), and its promoter locus presents binding sites for several transcription factors, including nuclear factor kappa B (NF-κB), IRF3/7, and c-myc (27, 28). Interestingly, in a kind of feedback mechanism, miR-146a targets two NF-κB signaling transducers, the tumor necrosis factor (TNF) receptor associated factor 6 (TRAF6) and the interleukin 1 receptor associated kinase 1 (IRAK1), which are key components of the MyD88-dependent TLR pathways (28). By targeting TRAF6 and IRAK1, miR-146a acts as potent inhibitor of TLR-mediated innate immune responses, preventing an overstimulation of the inflammatory response and ensuring immune tolerance (20, 28). In addition, miR-146a is able to regulate B-cell function and GC response. Indeed, its deficiency has been shown to promote the activation of c-REL, a NF-κB subunit implicated in B-cell proliferation, differentiation, and GC development (29, 30), which was reported to be a direct target of the miRNA in B cells (31). Moreover, as demonstrated in mice by Pratama et al. (32), miR-146a limits the accumulation of follicular T helper (Tfh) cells and GC B cells by targeting the inducible T-cell costimulator (ICOS) and its ligand (ICOSL), which are also critically involved in GC formation (33). Furthermore, enhanced miR-146a expression was associated with downregulation of Fas cell surface death receptor (FAS) in naïve B cells, unbalancing lymphocyte homeostasis and leading to hyper lymphoproliferation, and GC formation (34).

The ability of miR-146a to control TLR signaling and GC development makes it a good candidate to play a role in the intrathymic MG pathogenesis, since altered miR-146a expression could well-contribute to uncontrolled TLR activation and dysregulated B-cell function, which characterize hyperplastic MG thymuses.

Previous studies showed a significant upregulation of miR-146a in peripheral blood mononuclear cells (PBMCs) of MG patients compared to healthy controls (35), and clinical amelioration was observed in experimental autoimmune myasthenia gravis (EAMG) animals treated with antagomiR-146a (36). However, the possible contribution of miR-146a to autoimmunity development in MG thymus, and its perpetuation in peripheral blood, has never been thoroughly investigated.

In the present study, we performed a comprehensive analysis of miR-146a expression, along with that of its target genes, in hyperplastic thymuses and peripheral blood of MG patients. Our data revealed a possible role of miR-146a as key molecular link between intrathymic innate immunity and B-cell-mediated autoimmunity in MG.

## Methods

### Patients and Biological Samples

The study included 27 follicular hyperplastic thymuses from early-onset (<50 years) AChR-positive MG patients who underwent thymectomy as a therapeutic treatment and 10 non-pathological thymuses from patients without autoimmune diseases who underwent cardiovascular surgery (Table 1). Of the MG patients, 15 were treated with corticosteroids, and 12 were untreated or treated only with cholinesterase inhibitors at the time of thymectomy. MG thymuses were classified as follicular hyperplastic at the Department of Pathological Anatomy, Azienda Ospedaliera Bolognini (Seriate, Bergamo) according to the presence of at least one GC for thymic section: 5–15 GCs for section were present in thymuses from corticosteroid-naïve patients; 1–3 GCs for section were observed in corticosteroid-treated patients. For each thymus, some fragments were fixed in 10% formalin for histopathological classification; other fragments were snap frozen in optimal cutting temperature (OCT) and stored at −80 °C pending molecular and immunofluorescence analyses.

**Table 1:** Summary of the main features of acetylcholine receptor myasthenia gravis (AChR-MG) patients and controls included in the study.

	**Thymus**		**PBMCs and Serum**	
	**Normal (n = 10)**	**Hyperplastic MG (n = 27)**	**Healthy controls (n = 11)**	**MG patients (n = 31)^a^**
Sex (F:M)	4:6	23:4	7:4	21:10
Age at onset (years, mean ± SD)	–	26.5 ± 8.9	–	36.8 ± 15.4^b^
Age at thymectomy (years, mean ± SD)	26.0 ± 16.1	29.2 ± 8.1	–	–
Age at blood collection (years, mean ± SD)	–	–	33.4 ± 8.7	41.5 ± 14.5
Number of corticosteroid-treated patients	–	15	–	13

a *Serum was available for 21 of the 31 MG patients at the same time of PBMC collection (age at onset 38.0 ± 13.9; age at blood collection 43.3 ± 15.3)*.

b *Information on age at onset was not available in 6 of the 31 patients*.

Since serum and PBMCs from MG patients and controls included in the thymus analysis were not available, peripheral blood was collected from an independent group of 31 not thymectomized AChR-positive MG patients, of whom 13 were under treatment with corticosteroids, and from 11 healthy controls (Table 1), to collect PBMCs and serum. PBMCs were isolated by Lymphoprep (Axis-Shield, Dundee, Scotland) according to the manufacturer's instructions, frozen in fetal bovine serum (FBS) plus 10% dimethyl sulfoxide (DMSO) (Euroclone, Milan, Italy), and stored in liquid nitrogen until use.

None of the MG patients displayed any infectious diseases. The study was approved by the Ethics Committee of the Fondazione I.R.C.C.S. Istituto Neurologico Carlo Besta in Milan (Approval No. 586/2014), and each patient and control provided written informed consent for thymectomy and use of thymus specimens or blood for research purposes.

### Laser-Capture Microdissection

Seven of the 27 (Table 1) snap-frozen hyperplastic MG thymuses were subjected to laser-capture microdissection (LCM) of GCs using a Nikon Eclipse TE2000-S microscope (Nikon GMBH, Germany), equipped with a laser microdissector CellCut (MMI). For each thymus, six to ten 15-μm thick serial sections were mounted on membrane slides for LCM, stained by 50% hematoxylin and fixed in RNase-free 75–100% ethanol. Sections before and after the microdissected ones were stained for CD20, a B-cell marker, to identify GCs. From each MG thymic sample, at least 15 GCs (from consecutive serial sections) were microdissected and pooled in a single cap; sections devoid of the microdissected GCs were collected in separate caps. Whole sections from 4 of the 10 control thymuses (Table 1) were also collected.

The isolated tissue fragments of each series were incubated in lysis buffer (RNeasy Micro Kit, Qiagen, Valencia, CA) at 37 °C for 1 h and centrifuged at 800 × *g* for 5 min; lysates were then stored at −80 °C until use.

### *In vitro* Treatment of PBMCs With Prednisone

PBMCs isolated from four MG patients corticosteroid-naïve at time of bleeding and from three healthy controls were plated in a 48-well plate (0.5 × 10^6^ cells/well) in RPMI 1640 supplemented with 10% heat-inactivated FBS, 1% penicillin/streptomycin, 1% glutamine, 1% non-essential amino acids, 1% sodium pyruvate, 0.1% β-mercaptoethanol, and 0.1% concanavalin A, and kept at 37 °C in a humidified 5% CO_2_ atmosphere for 24 h. Cells were treated with a non-toxic dose of 0.1 μM prednisone (Sigma-Aldrich, Darmstadt, Germany) (37), for 3 days, collected in TRIzol after 6, 24, 48, and 72 h of treatment and stored at −80 °C for RNA extraction. Concanavalin A was added to the medium as lymphocyte mitogen, for allowing PBMCs to proliferate and avoiding spontaneous cell death without stimuli up to 72 h of prednisone treatment. This was important to test the impact of the drug without bias due to spontaneous cell death. Concanavalin A, or other mitogens, are widely used in *in vitro* studies performed on PBMCs, such as studies exploring PBMC response to prednisone or other corticosteroid drugs (37–39). Cell viability was evaluated by Countess II Automated Cell Counter (Thermo Fisher Scientific, Waltham, MA).

### RNA Isolation

Total RNA was extracted from frozen thymic fragments and treated and untreated PBMCs using the TRIzol method (Thermo Fisher Scientific), previously reported to efficiently recover miRNAs and showing comparable results, in different tissue samples, to those obtained using specific kits for miRNA enrichment (e.g. MirVana kit) (40, 41). To note, the use of this kind of kits is considered optimal for miRNA isolation when unbiased high-throughput approaches are used downstream, since they allow detection of different miRNAs with the same efficiency, avoiding the risk to lose some molecules (41). Here, we aimed at assessing the expression of one candidate miRNA, miR-146a, along with its messenger RNA (mRNA) target genes, and obtained evidence of efficient miR-146a amplification in TRIzol-extracted RNA samples using the TaqMan real-time PCR protocol described below.

For LCM samples and sera, we used the RNeasy Micro Kit and the miRNeasy Serum/Plasma Kit (Qiagen), respectively. All extractions were performed according to manufacturer's instructions. Quality and concentration of RNA were evaluated by a NanoDrop 2000 spectrophotometer (Thermo Fisher Scientific).

### Reverse Transcription and Real-Time PCR

Total RNA samples were reverse transcribed using a TaqMan MicroRNA Reverse Transcription Kit with primers specific for miR-146a and for human small nuclear RNA (snRNA) U6, included as endogenous control. Complementary DNAs (cDNAs) (corresponding to 15 ng total RNA) were amplified in duplicates by quantitative real-time PCR, using predesigned TaqMan MicroRNA assays, on ViiA7 Real-Time PCR system (Thermo Fisher Scientific). For gene expression analyses, cDNA samples were prepared from total RNA using Superscript VILO cDNA Synthesis Kit (Thermo Fisher Scientific) and subjected to real-time PCR reactions in duplicates with predesigned functionally tested TaqMan gene expression assays specific for IRAK1, TRAF6, c-REL, ICOS, FAS, and interferon regulatory factor 8 (IRF8) (Thermo Fisher Scientific). Human 18S was used as endogenous control for the normalization of gene expression data (Thermo Fisher Scientific). U6 and 18S were stably expressed across control and the MG samples (normally distributed Ct values with SD ≤ 0.5). Transcriptional levels of miR-146a and target genes were expressed as relative values normalized with U6 or 18S, respectively, using the formula 2^−ΔCt^ × 100.

### Double Immunofluorescence

Double immunofluorescence stainings were performed on 6-μm thick serial sections of snap-frozen follicular hyperplastic thymuses from five corticosteroid-naïve and four corticosteroid-treated MG patients and six control thymuses. Sections were fixed in 4% paraformaldehyde (PFA) for 10 min and incubated in cold methanol for 10 min and in 5% bovine serum albumin (BSA) for 1 h; then, they were immunostained overnight at 4 °C with a combination of primary antibodies against IRAK1 (1:50, rabbit polyclonal, Thermo Fisher Scientific), c-REL (1:50, rabbit polyclonal, Thermo Fisher Scientific), CD20 (1:300, clone L26, Agilent Dako, Santa Clara, CA), CD11c (1:20, clone B-ly6, BD Pharmigen, San Jose, CA), cytokeratin (CK) (1:100, clone MNF116, Agilent Dako), and CD68 (1:100, clone KP1, Agilent Dako). Sections were then incubated for 1 h with a mixture of Cy2-conjugated goat antimouse immunoglobulin G (IgG) and Cy3-conjugated goat antirabbit IgG (Jackson ImmunoResearch Laboratories, West Baltimore Pike, West Grove, PA); nuclei were stained with 4′,6-diamidino-2-phenylindole (DAPI; Thermo Fisher Scientific). As negative control, primary antibodies were omitted or replaced with isotype-specific IgGs (Agilent Dako). Fluorescence images were captured by the C1 laser scanning confocal microscope system (Nikon) and analyzed using Image J software (version 1.48). CD20/IRAK1, CD11c/IRAK1, and CD68/IRAK1 double-positive cells were counted by two experienced operators blinded to the diagnosis and treatment on at least four randomly selected adjacent fields per section at ×60 magnification.

### Statistical Analysis

The non-parametric distributed data, tested via Shapiro–Wilk test, were analyzed by Kruskal–Wallis test with Dunn's *post-hoc* test for multiple comparisons or by Mann–Whitney test for comparison of two groups, as indicated in figure legends. Differences were considered statistically significant when the *p* values were < 0.05. Nonparametric Spearman correlation test was applied to evaluate the correlation between miR-146a and its target expression levels in thymic tissues. Receiver operating characteristic (ROC) curves were used to assess the sensitivity and specificity of miR-146a in serum as biomarker for MG. GraphPad Prism v5.0 (La Jolla, CA) was used for data elaboration and statistical analyses.

## Results

### miR-146a Deficiency in Follicular Hyperplastic MG Thymus

**Figure 1:**
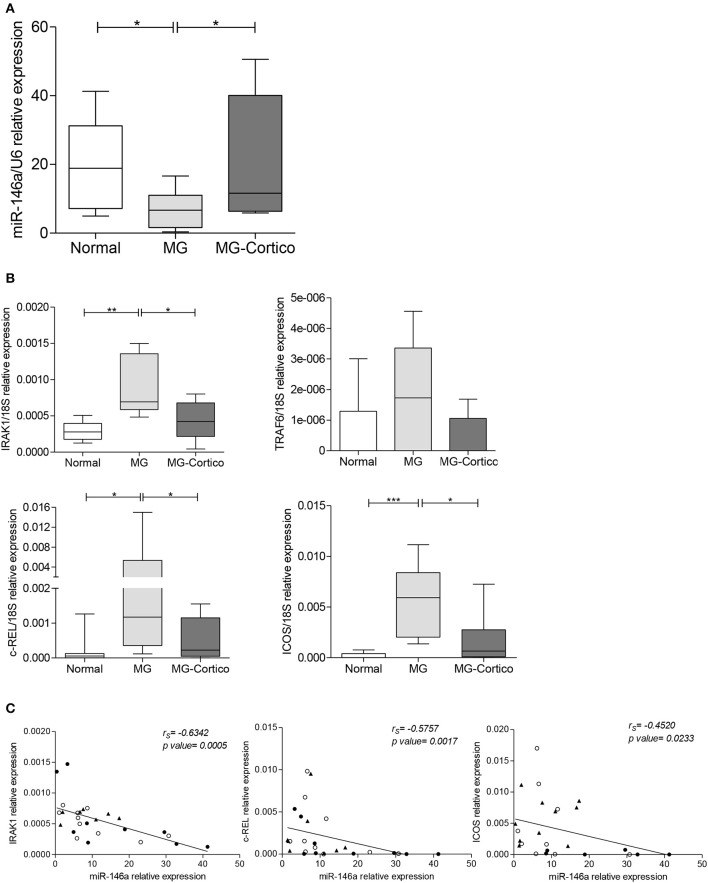
Decreased expression of miR-146a in follicular hyperplastic myasthenia gravis (MG) thymuses and increased transcriptional levels of its target genes, IRAK1, TRAF6, c-REL, and ICOS. **(A)** Real-time PCR analysis of miR-146a levels in normal thymuses (*n* = 10) and follicular hyperplastic thymuses from MG patients classified based on treatment before thymectomy in untreated or treated only with cholinesterase inhibitors (*n* = 12, MG) and treated with corticosteroids (*n* = 15, MG-Cortico). **(B)** Real-time PCR analysis to assess the expression of miR-146a gene targets IRAK1, TRAF6, c-REL, and ICOS in the same normal and MG thymuses. In **(A,B)** boxplots, miR-146a and target expression levels were expressed as relative values (2^−ΔCt^ × 100) normalized toward the endogenous small nuclear RNA (snRNA) U6 (miR-146a) or 18S (target genes); dark horizontal lines represent means, with the box representing the 25 and 75th percentiles and the whiskers the 5 and 95th percentiles. *p* values were assessed by the Kruskal–Wallis test followed by the Dunn's *post hoc* test, *p < 0.05; **p < 0.01; ***p < 0.001. **(C)** Negative correlation estimated by Spearman's correlation analysis between miR-146a levels and messenger RNA (mRNA) levels of IRAK1, c-REL, and ICOS in the thymus of 10 controls (•), 12 corticosteroid-naïve (°), and 15 corticosteroid-treated (▴) MG patients.

The expression of miR-146a was assessed in follicular hyperplastic thymuses from early-onset AChR-positive MG patients and normal thymuses from patients without autoimmune diseases. Patients were stratified in corticosteroid-naïve and corticosteroid-treated patients according to the therapeutic treatment before thymectomy. The first group was characterized by a higher number of GCs per thymic section compared to the second one (5–15 vs. 1–3 GCs), in line with the corticosteroids' ability to reduce thymic GCs in MG patients (42). Interestingly, a significant lower expression of miR-146a was observed in hyperplastic thymuses from corticosteroid-naïve MG patients compared to normal thymuses (Figure 1A). This decrease was not found in thymuses from corticosteroid-treated patients, suggesting that corticosteroids may affect miR-146a expression (Figure 1A). No significant difference in the intrathymic miRNA levels was found between male and female patients and controls (data not shown). We then analyzed the transcriptional levels of IRAK1, TRAF6, c-REL, and ICOS, well-known miR-146a targets critically involved in innate immune response activation and GC formation (20, 28, 30–32), which are key events in the intrathymic MG pathogenesis (4, 5). IRAK1, c-REL, and ICOS mRNA levels showed an opposite trend compared to that of miR-146a: they were increased in thymuses of corticosteroid-naïve patients compared to both immunosuppressed MG patients' and normal thymuses (Figure 1B). These results supported an effect of corticosteroid therapy on the miR-146a/mRNA target axis. Regarding the expression of TRAF6, although difference among the sample groups did not reach statistical significance, corticosteroid-naïve hyperplastic MG thymuses showed increased mRNA levels of this gene, as observed for the other miR-146a targets (Figure 1B).

A negative correlation was found between miR-146a levels and the mRNA levels of IRAK1, c-REL, and ICOS in MG and control tissues (Figure 1C), in accordance with the existence of a functional relationship between the miRNA and its three target genes (20, 28, 30–32).

### miR-146a Expression in Germinal Centers of Hyperplastic MG Thymuses

**Figure 2:**
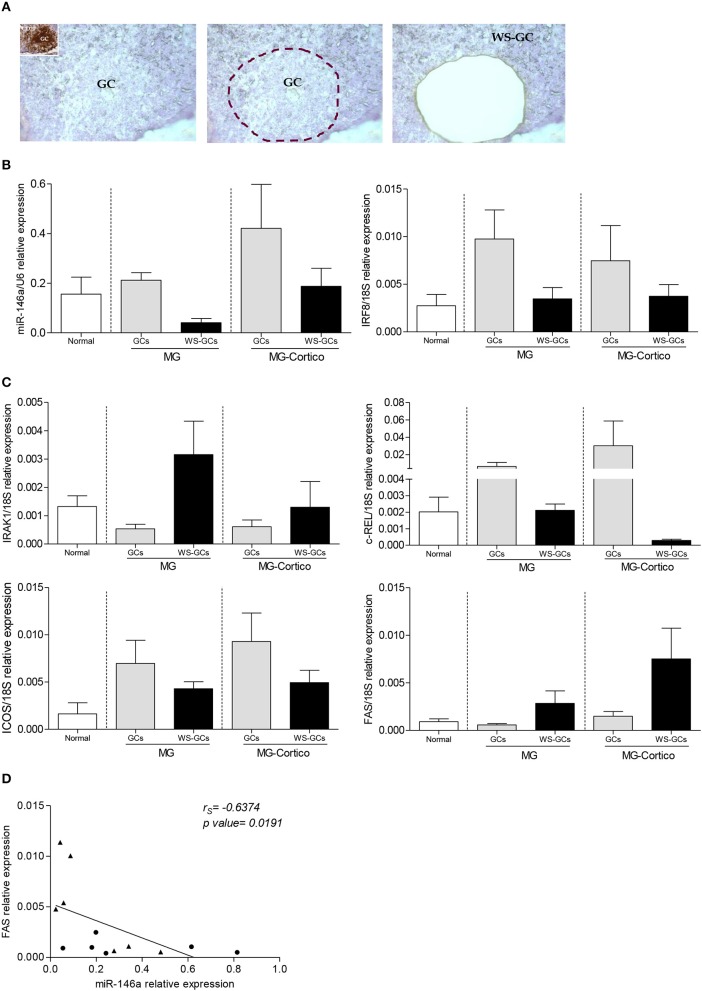
Analysis of miR-146a and its target gene messenger RNA (mRNA) levels in microdissected germinal centers (GCs) of hyperplastic myasthenia gravis (MG) thymuses. **(A)** Representative image showing a follicular hyperplastic MG thymic section containing a GC subjected to laser capture microdissection. The inset in the first panel corresponds to an adjacent section of the same thymus stained for the B-cell marker CD20. Expression of miR-146a and IRF8 **(B)** and miR-146a targets IRAK1, c-REL, ICOS, and FAS **(C)** in normal thymus sections (*n* = 4), whole thymic sections devoid of GCs (WS-GCs), and the corresponding microdissected GCs, from corticosteroid-naïve (*n* = 4, MG) and corticosteroid-treated (*n* = 3, MG-Cortico) MG patients. In the graphs, miR-146a, IRF8, and miRNA target gene levels were expressed as relative values (2^−ΔCt^ × 100) normalized toward the endogenous small nuclear RNA (snRNA) U6 (miR-146a) or 18S (IRF8 and target genes). **(D)** Negative correlation estimated by Spearman's correlation analysis between miR-146a levels and mRNA levels of FAS in GCs (•) and WS-GCs (▴) from hyperplastic MG thymuses.

Based on the hypothesis that miR-146a/mRNA target changes observed in MG thymuses could be related to GC presence and to structural changes in GC organization during corticosteroid treatment, we next compared miR-146a, along with IRAK-1, c-REL, and ICOS expression, in microdissected GCs, and the corresponding GC-surrounding tissue (WS-GCs), from follicular hyperplastic MG thymuses (Figure 2A) and whole sections from normal thymuses. To control GC microdissection quality, the IRF8 transcript was analyzed in all the samples as GC marker (43, 44). As expected, IRF8 mRNA levels were higher in microdissected GCs, both from corticosteroid-naïve and corticosteroid-treated patients, compared to WS-GC sections of follicular hyperplastic thymuses and whole thymic sections from controls (Figure 2B). In pathological thymuses from untreated MG patients, miR-146a was expressed at lower levels in WS-GC tissues, but it was expressed in GCs, indicating that the miR-146a deficiency observed in hyperplastic MG thymuses was not directly related to GC presence. Indeed, miR-146a levels did not correlate with the GC number in the hyperplastic thymic sections (data not shown). In corticosteroid-treated patients, the miRNA levels were higher in both GC and WS-GC tissue, compared to the levels observed in corticosteroid-naïve patients, in line with the hypothesis that corticosteroids influence miR-146a expression (Figure 2B). IRAK1 showed the opposite expression trend of miR-146a: its transcriptional levels were higher in WS-GCs, and lower in GCs, in untreated patients' thymuses compared to normal thymuses; moreover, they were reduced in the WS-GCs of immunosuppressed patients (Figure 2C). This suggested that defective miR-146a expression in the hyperplastic thymic medulla, out of GCs, might lead to IRAK1 overexpression; in addition, miR-146a induction by the immunosuppressive treatment seems to normalize/reduce IRAK1 expression. As regard c-REL and ICOS, the increased expression levels previously found in hyperplastic MG thymuses (Figure 1B) were due to GC presence. Indeed, microdissected GCs from untreated and treated patients showed the highest c-REL and ICOS levels (Figure 2C). In corticosteroid-treated patients, lower expression of c-REL was observed in WS-GCs compared to the same tissues from untreated patients and normal thymuses, in line with the increased expression of miR-146a in WS-GCs.

Based on the literature data showing a contribution of miR-146a to GC formation via FAS (34), we also analyzed FAS expression in the microdissected samples. In untreated MG patients, FAS mRNA levels were lower in GCs than in the corresponding WS-GCs (Figure 2C), sustaining the hypothesis of a miR-146a contribution to GC formation via Fas downregulation in B cells. In corticosteroid-treated patients, WS-GCs presented higher levels of FAS mRNA compared to the thymic samples of untreated patients and controls, indicative of an impact of the corticosteroid treatment in inducing programmed cell death in thymic cell populations (Figure 2C).

In line with previous data showing that FAS is a direct target of miR-146a in B cells (34), we found a negative correlation between miR-146a and FAS mRNA levels in GCs and WS-GCs from MG thymuses (Figure 2D).

### Increased Expression of IRAK1 in Myeloid Dendritic Cells and Macrophages of Hyperplastic MG Thymuses

**Figure 3:**
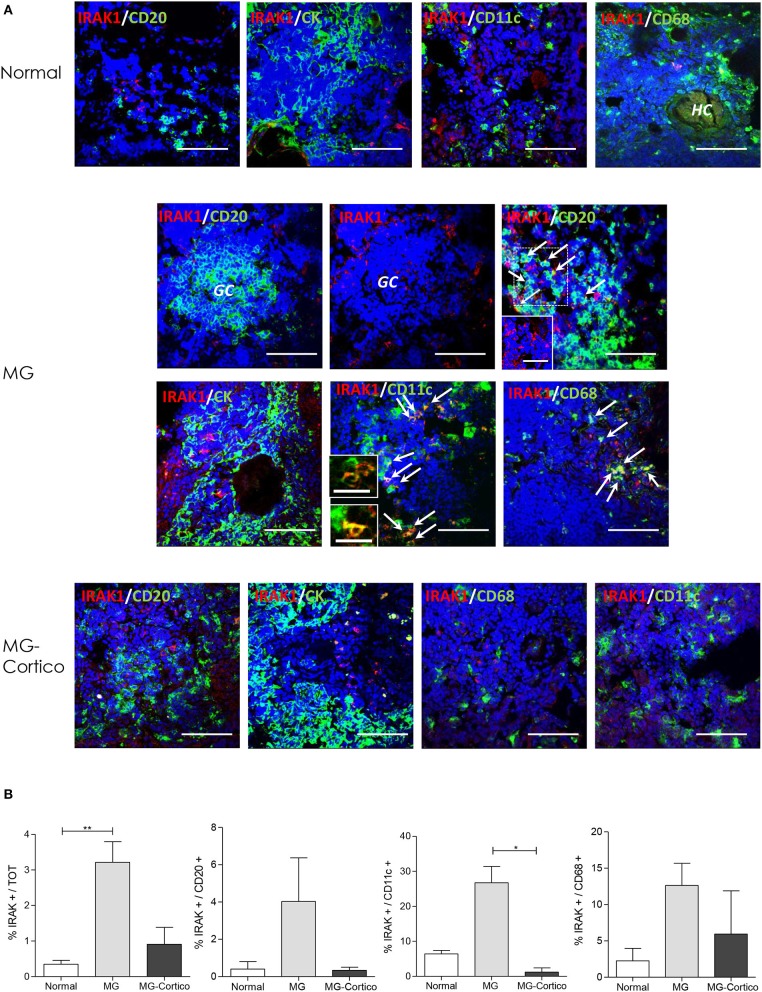
Increased expression of interleukin 1 receptor associated kinase 1 (IRAK1) in infiltrating B cells, myeloid dendritic cells (mDCs), and macrophages of hyperplastic myasthenia gravis (MG) thymuses. **(A)** Double immunofluorescence stainings of normal thymus (upper panels) and hyperplastic MG thymuses from corticosteroid-naïve (MG) and corticosteroid-treated (MG-Cortico) MG patients (middle and lower panels) for the expression of IRAK1 (red) in combination with: CD20 (green), marker for B cells; cytokeratin (CK, green), marker of epithelial cells; CD11c (green), marker for mDCs; and CD68 (green), marker for macrophages. The arrows in the middle panels indicate double-positive cells. Blue staining shows 4′,6-diamidino-2-phenylindole (DAPI)-positive nuclei. The inset in MG IRAK-1/CD20 panel shows cells stained only for IRAK1 present in the main panel (dashed box). The insets in MG IRAK1/CD11c panel show enlargement of mDCs cells expressing IRAK1, present in the main panel. HC, Hassall's corpuscle. Magnification bars: 50 μm in the main panels and in MG and MG-Cortico IRAK1/CD20 insets; 20 μm in the MG IRAK1/CD11c insets. **(B)** Mean percentage (±SEM) of IRAK1-positive cells estimated on total DAPI-positive cells, total CD20-positive B cells, total CD11c-positive mDCs, and total CD68-positive macrophages in six control thymuses, five corticosteroid-naïve (MG), and four corticosteroid-treated (MG-Cortico) MG patients (four adjacent fields for sample group). *p* values were assessed by the Kruskal–Wallis test for multiple comparisons followed by the Dunn's *post-hoc* test, *p < 0.05; **p < 0.01.

As described above, in follicular hyperplastic MG thymuses, IRAK1 expression was increased in medullary area out of GCs, indicating that other thymic cell populations than GC cells overexpressed this molecule in the pathological tissues. Since IRAK1 is a critical component of TLR signaling pathways, we investigated its expression in thymic cells, known to overexpress TLRs (i.e. TLR4, TLR7, and TLR9) (10, 11), including CK-positive thymic epithelial cells (TECs), CD11c-positive myeloid dendritic cells (mDCs), and CD68-positive macrophages. CD20-positive B cells were included in the analysis since diffuse B-cell lymphoid infiltrates consistently characterize medulla of hyperplastic MG thymuses (9, 11). By double immunofluorescence, we observed IRAK1 expression in a proportion of infiltrating B cells of hyperplastic MG thymuses from untreated patients, but scarcely in GCs and B-cell infiltrates of pathological tissues from corticosteroid-treated patients and B cells of normal thymuses (Figure 3A). Indeed, although differences did not reach statistical significance, the percentage of diffuse CD20-positive B cells expressing IRAK1 was higher in thymuses from untreated MG patients compared to control and corticosteroid-treated MG patients' thymuses (Figure 3B). MG and control thymuses did not show marked difference in IRAK1 expression in TECs (Figure 3A). On the contrary, the protein expression in mDCs and macrophages was increased in thymuses from untreated MG patients compared to controls and tissues from immunosuppressed patients, in terms of both intensity of the immunostaining and number of positive cells (Figures 3A,B).

By considering IRAK1-positive cells, irrespective of the phenotype, the immunofluorescence analysis showed an increased percentage of these cells in the thymus of corticosteroid-naïve MG patients compared to control thymuses (Figure 3B). According to molecular data, this percentage was reduced in thymuses from patients treated with corticosteroids before thymectomy, supporting a corticosteroid effect in IRAK1 normalization within the MG thymus, which could occur via miR-146a.

### Marked c-REL Expression in Germinal Center and Infiltrating B Cells of MG Thymuses

**Figure 4:**
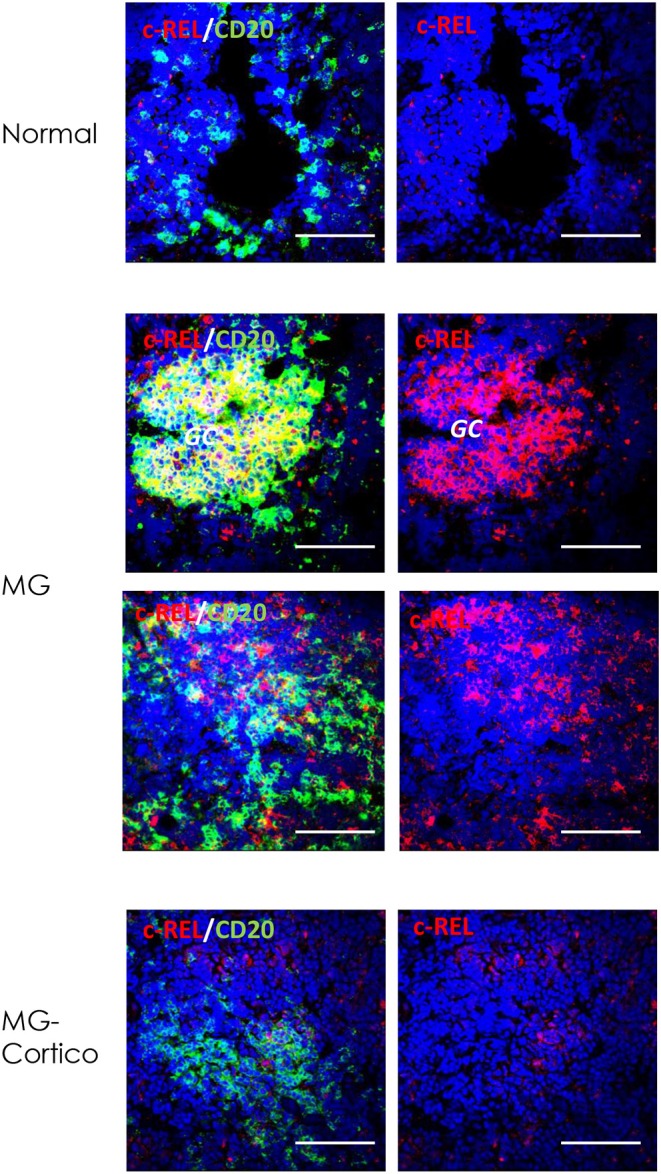
Immunoreactivity for c-REL of germinal center (GC) and infiltrating B cells of hyperplastic myasthenia gravis (MG) thymuses. Double immunofluorescence stainings of normal thymus (upper panels) and hyperplastic MG thymic sections from untreated (MG) and corticosteroid-treated (MG-Cortico) patients (middle and lower panels) for c-REL (red), alone (right panels) or in combination with CD20 (green) (left panels), marker of B cells. Blue staining shows DAPI-positive nuclei. Magnification bars: 50 μm in all panels.

By immunofluorescence, we found a strong immunoreactivity for c-REL of follicular hyperplastic thymuses from corticosteroid-naïve MG patients compared to normal thymuses. This immunoreactivity was marked in GCs, highlighting the role of c-REL in GC formation (Figure 4). In addition, enhanced c-REL expression was observed in infiltrating B cells of the thymic medulla in untreated MG patients, indicating that increased levels of c-REL in hyperplastic MG thymuses were mainly due to GC and infiltrating B cells. In contrast, reduced c-REL expression was observed in thymuses of patients treated with corticosteroids before thymectomy (Figure 4), suggesting a possible impact of immunosuppressive drugs on GCs and infiltrating B cells via the miR-146a/c-REL axis.

### Dysregulated Expression of miR-146a in Serum and PBMCs of MG Patients

**Figure 5:**
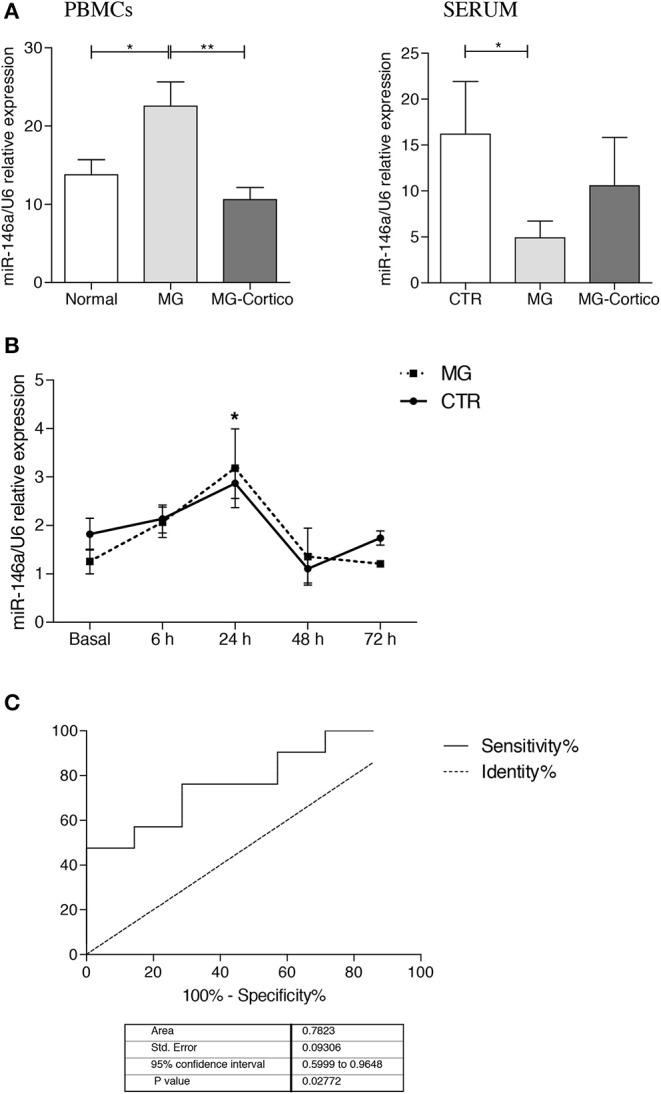
Peripheral expression of miR-146a in myasthenia gravis (MG). **(A)** Real-time PCR analysis of miR-146a expression in peripheral blood mononuclear cells (PBMCs) (left graph) and serum (right graph) from MG patients (*n* = 31, 13 of whom were corticosteroid-treated, MG-Cortico) and healthy controls (*n* = 11). miR-146a levels were expressed as relative values (2^−ΔCt^ × 100) normalized toward the endogenous small nuclear RNA (snRNA) U6. *p* values were assessed by Kruskal–Wallis test followed by the Dunn's *post hoc* test. *p < 0.05; **p < 0.01. **(B)** *In vitro* assay of miR-146a expression in PBMCs from four MG patients and three healthy controls, treated with prednisone at 6, 24, 48, and 72 h. miR-146a levels were analyzed by real-time PCR and expressed as relative values (2^−ΔCt^ × 100) normalized toward U6 snRNA. In the graph, the mean relative expression levels ± SEM of miR-146a estimated in cells from the four MG patients and from the three controls at basal condition and after 6, 24, 48, and 72 h of treatment with prednisone are shown. *p* values were assessed by Kruskal–Wallis test followed by the Dunn's *post hoc* test. *p < 0.05 (24 h of treatment vs. basal condition). **(C)** Receiver operating characteristic (ROC) curves used to assess the sensitivity and specificity of miR-146a in serum as biomarker for MG.

To check for possible miR-146a dysregulation also in peripheral blood of MG patients, we analyzed the miRNA expression in serum and PBMCs of a cohort of MG patients and healthy controls (Table 1) by real-time PCR. We found a significant downregulation of miR-146a in the serum of corticosteroid-naïve MG patients compared to healthy controls (Figure 5A), as observed in the thymus, whereas in corticosteroid-treated patients, serum miR-146a levels were comparable to those of controls. In line with previous literature data (35), we found that miR-146a expression was significantly increased in PBMCs of corticosteroid-naïve patients compared to controls, suggesting a possible defective miR-146a release in serum by PBMCs. miR-146a increase in PBMCs was not observed in immunosuppressed patients (Figure 5A), again indicating differences between untreated patients and patients under immunosuppressive therapy. Thus, to understand the effect of corticosteroids on miR-146a expression, we performed *in vitro* treatment of PBMCs from corticosteroid-naïve MG patients and healthy controls with prednisone for 6, 24, 48, and 72 h. We did not observe differences in miR-146a levels at 0, 6, 24, 48, and 72 h between MG and control PBMCs. In both MG and control cells, we observed that the drug was able to increase miR-146a levels with a peak at 24 h of treatment (Figure 5B). These data were in line with the increased expression levels of miR-146a observed in thymuses from corticosteroid-treated compared to untreated patients. Lower expression of miR-146a in PBMCs of corticosteroid-treated compared to corticosteroid-naïve patients could be explained by considering that corticosteroids may induce miR-146a expression, but this induction might be accompanied by an increased release of the miRNA in serum. Indeed, serum levels of miR-146a were higher in patients under immunosuppressive therapy compared to untreated patients (Figure 5B).

Potential value of serum miR-146a as biomarker for MG was evaluated by ROC curve analysis. Of interest, in our cohort of AChR-positive patients and controls, we obtained sensitivity and specificity performance results that suggested a possible role of miR-146a as disease biomarker for AChR-MG (Figure 5C): area under curve (AUC) was 0.782 (95% CI, 0.5999–0.9648) (*p* = 0.02).

## Discussion

MiRNA dysregulation is critically involved in the development of several autoimmune diseases (18, 19, 45). A number of studies showed alterations of miRNAs in PBMCs and serum of myasthenic patients, suggesting a key role of these molecules also in the immune pathophysiology of MG (46–50). The contribution of miRNAs to the intrathymic MG pathogenesis has been poorly explored. A recent miRNome profiling performed in hyperplastic MG thymuses highlighted the role of specific miRNAs (e.g. miR-7 and miR-125a) in thymic changes, as well as inflammatory pathways and immune dysregulation, associated with MG (51). miR-146a is one of the first miRNAs identified to be involved in the modulation of innate and adaptive immune system, whose role in autoimmunity has been reported in different studies (20–24). Physiologically, it is induced by TLR ligands in an NF-κB-dependent manner and acts as inhibitor of innate immune responses by targeting IRAK1 and TRAF6, two key effectors of TLR signaling (20). Its ability to control TLR pathways allows inflammation resolution, avoiding persisting inflammatory reactions that could be dangerous and favor chronic inflammatory and autoimmune conditions (20, 28). At the same time, miR-146a has been reported to participate to GC development, by targeting c-REL, ICOS, and FAS (30–32, 34).

Chronic inflammation, abnormal TLR activation, and GC formation characterize hyperplastic thymus of patients affected by MG (5, 8), but the contribution of miR-146a to the intrathymic MG pathogenesis has not been deeply investigated. In the present study, we analyzed the expression of miR-146a, and its above-mentioned targets, in follicular hyperplastic MG thymuses and normal thymuses from patients without autoimmune diseases. Interestingly, we provided evidence of dysregulated miR-146a/mRNA target pattern in MG thymuses. Indeed, we showed that miR-146a expression was defective in hyperplastic thymus of patients untreated, or treated only with cholinesterase inhibitors before thymectomy, compared to controls. This deficiency was associated with a significant increased expression of IRAK1, c-REL, and ICOS, whose expression levels negatively correlated with those of miR-146a, supporting a functional miR-146a/mRNA target relationship. Considering the critical role of IRAK1 in TLR signaling (20, 28), and of c-REL and ICOS in GC formation (29, 33), these results strongly suggested that miR-146a downregulation could significantly contribute to persistent innate immune activation, sustained inflammation, and follicular hyperplastic changes in the thymus of MG patients. In line with this hypothesis, gene knockout studies, aimed at investigating the miR-146a function, showed that deficiency of this miRNA may lead to an excessive IL-6 and TNF-α production, hyperresponsiveness to bacterial lipopolysaccharide (LPS), chronic inflammation, and spontaneous autoimmunity (52, 53). Not surprisingly, miR-146a-deficient expression has been described in other autoimmune conditions, than MG. In particular, miR-146a levels were found to be lower in PBMCs and serum of patients with SLE compared to controls (21, 54). Underexpression of miR-146a in these patients was shown to contribute to activation of type I IFN pathways, and its overexpression reduced the induction of these pathways in PBMCs (55), also suggesting that the miR-146a decrement that we observed in MG thymuses could favor type I IFN overproduction, which is an intrathymic hallmark of MG patients (56). Interestingly, in a study by Rosato et al. (57), low miR-146a expression, correlating with elevated levels of IRAK1 and type I IFNs, was associated with Epstein–Barr virus (EBV) infection in cells expressing the EBV-encoded EBNA2, thus suggesting a possible relationship between the observed miR-146a reduction and the EBV presence that we previously found in hyperplastic MG thymuses (9). Moreover, decreased expression of miR-146a has been reported to contribute to abnormal regulatory T cell (Treg) phenotype in RA patients with active disease, and correlated with joint inflammation (58). Likewise, defective Tregs in hyperplastic MG thymuses (59) could be related to miR-146a decrease, a hypothesis to be deeply explored. Indeed, miR-146a is one of the miRNAs prevalently expressed in Tregs and is critical for their suppressor function by targeting the signal transducer and activator transcription 1 (STAT1), so that its deletion may result in tolerance breakdown (60).

Of note, the analysis of miR-146a/mRNA target expression in hyperplastic thymuses from MG patients treated with corticosteroids before thymectomy revealed that the immunosuppressive therapy was able to normalize the intrathymic miRNA levels, along with the transcriptional levels of IRAK1, c-REL, and ICOS. Indeed, miR-146a expression was increased in immunosuppressed compared to untreated patients. This finding was in line with previous observations demonstrating that miR-146a is a glucocorticoid-inducible miRNA, along with mir-26b, mir-125a-5p, mir-150-5p, and mir-184 (61). Accordingly, normalization of IRAK1, c-REL, and ICOS mRNA levels in corticosteroid-treated patients was likely due to miR-146a restoration, as an effect of the therapy. However, corticosteroids are potent modulators of immune system with a strong impact on immune system cells and ability to modify miRNA pattern at different levels; thus, their effects on miR-146a expression could be both direct or indirect. Since immunosuppressive treatment is known to affect the number of GCs in the MG thymus (42), we also hypothesized that differences in thymic miR-146a/mRNA target pattern between corticosteroid-naïve and corticosteroid-treated patients could be related to GC changes upon treatment. However, by performing LCM experiments, we demonstrated that miR-146a deficiency in hyperplastic MG thymuses was not related to GCs. Indeed, in untreated patients, miR-146a was downregulated in the GC-surrounding medulla, where IRAK1 was upregulated, but it was expressed in GCs. Moreover, its levels did not correlate with the GC number in the MG thymuses. In treated patients, miR-146a expression was higher in both GCs and WS-GCs compared to untreated patients, again supporting an effect of corticosteroids in inducing the miRNA expression. Our data also highlighted the role of c-REL and ICOS in GC formation and maintenance in MG thymuses. Indeed, c-REL and ICOS were expressed at higher levels in GCs of both untreated and corticosteroid-treated patients than in control thymuses. Of interest, in MG GCs and WS-GCs, a negative relationship was found between miR-146a expression and mRNA levels of FAS, another recognized direct target of miR-146a (34), indicating a key contribution of the miRNA to GC formation via FAS downregulation. The role of FAS in GCs is strongly supported by studies performed in B-cell-specific FAS-deficient mice, found to develop fatal lymphoproliferation due to B-cell activation, and by the observation that ablation of FAS specifically in GC B cells reproduced lymphoproliferation (62). Downregulation of FAS in GCs by miR-146a was also reported to cause autoimmune lymphoproliferative syndrome in mice (34), which is indicative of a critical role of miR-146a in B-cell homeostasis and GC response through FAS. Consistent with these observations, Cho et al. demonstrated that not only elevated levels of miR-146a in B cells are important in controlling humoral autoimmunity by targeting CD40 signaling pathways but also that specific deletion of miR-146a in T cells increases Tfh cell number enhancing GC reactions (31), thus sustaining that miR-146a expression is required for maintenance of GC reactions.

In line with LCM data, by double immunofluorescence, we found that IRAK1 was not expressed in GCs, but mainly in MG mDCs and macrophages, thymic cell populations characterized, in MG patients, by overexpression of TLRs, particularly TLR4, as reported in our previous studies (10, 11). Literature data showed that miR-146a efficiently targets TRAF6 and IRAK1 in DCs and modulates the production of pro-inflammatory cytokines by these cells (63). Thus, miR-146a deficiency observed in the medulla of hyperplastic MG thymuses could well-contribute to IRAK1 upregulation and TLR-mediated inflammatory activation in mDCs and macrophages. B cells of GCs, along with B-cell lymphoid infiltrates, were markedly labeled for c-REL, strengthening the role of this molecular factor in GC development and B-cell dysregulation in MG thymuses. Heise et al. (29) reported that GC B-cell-specific deletion of c-REL led to the collapse of established GCs and was associated with the failure to activate a metabolic program that promotes cell growth, unequivocally demonstrating the role of c-REL in GCs. Along with c-REL, ICOS, a recognized target of miR-146a that we found to be overexpressed in MG thymuses, actively participate in GC formation (32). Its blockage has been found to prevent Tfh and GC B-cell accumulation (32), suggesting that miR-146a break may significantly promote or enhance GC response also via ICOS in MG thymus.

Unfortunately, prethymectomy serum and PBMCs from patients included in the thymus analysis were not available. However, to verify whether miR-146a dysregulation also characterized MG peripheral blood, we investigated the miRNA expression in serum and PBMCs of a group of not thymectomized MG patients and controls. In untreated patients, miR-146a was downregulated in serum, as observed in SLE patients (54), but it was upregulated in PBMCs compared to controls, as previously reported in the literature for MG patients (35). Increased miR-146a levels, positively correlating with levels of proinflammatory cytokines, were reported in PBMCs, and particularly CD4-positive T cells, from patients with RA (22, 64). In an independent study, higher miR-146a levels were found in IL-17-producing T cells from RA patients with high disease activity, suggesting a role of miR-146a in the differentiation of Th17 cells (65). Similarly, by considering the key function of miR-146a in controlling Treg activity (60), the dysregulated miR-146a expression that we found in the MG peripheral blood could well-affect the T-cell inflammatory phenotype, contributing to the Th17/Treg unbalance characteristic of MG patients (66). miR-146a overexpression in PBMCs was also demonstrated in MS patients, and again, it was associated with increased Th1/Th17 cytokine overexpression (e.g. IL-17, IFN-γ, TNF-α) (67). Variation in serum and cellular miR-146a levels in an opposite manner led us to hypothesize a possible defective release of the miRNA from PBMCs to serum in MG patients. However, the exact mechanisms underlying miR-146a dysregulation in MG blood, as well as the miRNA impact on circulating immune system cells, needs to be further explored.

In corticosteroid-treated patients, PBMC levels of miR-146a were comparable to those of controls, again revealing an effect of immunosuppressive treatment on miR-146a expression. By *in vitro* experiments, we observed that treatment of PBMCs with prednisone was able to increase the expression of miR-146a in both MG and control cells, according to the already reported role of corticosteroids to induce the expression of this miRNA (61). Hence, this increase may explain normalization of miR-146a levels in hyperplastic MG thymuses of patients treated with corticosteroids before thymectomy. Since serum, but not PBMC, levels of miR-146a were higher in treated compared to untreated MG patients, we hypothesized that immunosuppressive drugs might induce miR-146a expression in PBMCs accompanied by high release in the serum. In addition, corticosteroids could affect viability of specific cell populations expressing the miRNA in PBMCs or thymus of treated patients; thus, their exact impact on miR-146a expression needs to be deeply studied.

Finally, ROC curve analyses provided sensitivity and specificity results indicative of a possible role of miR-146a in serum as biomarker for MG, whose usefulness for monitoring the disease progression or prognosis deserves future investigation. However, since the miRNA was widely implicated in other inflammatory and autoimmune pathologies (21–26), it could represent not a biomarker specific for MG, but a more general marker of an inflammatory autoimmune condition, with potential utility in more than one autoimmune disease.

Our overall findings thus revealed that miR-146a expression is defective in follicular hyperplastic MG thymuses and that loss of fine regulation of innate and adaptive immune response by the miRNA may significantly contribute to the intrathymic MG pathogenesis. The ability of miR-146a to control TLR signaling pathways, and consequently inflammation, along with GC formation, makes the miR-146a/mRNA target axis a promising candidate target of innovative treatments for counteracting B-cell-mediated autoimmunity in MG.

## Data Availability Statement

The datasets generated for this study are available on request to the corresponding author.

## Ethics Statement

The study was approved by the Ethics Committee of the Fondazione I.R.C.C.S. Istituto Neurologico Carlo Besta (Milan, Italy; Approval No. 586/2014). All patients and controls signed an informed consent form for the use of biological samples for research purpose.

## Author Contributions

FB participated in sample collection, performed immunofluorescence analyses and cell counting, elaborated the experimental data, performed statistical analysis, and drafted the manuscript. LS was involved in sample collection and molecular analyses and performed the *in vitro* experiments. SM contributed to the study design and participated in data discussion and elaboration. SB and RF performed clinical data collection and were involved in data discussion. TM contributed to collection of thymic samples and performed histological classification of MG thymuses. CA and RM provided clinical data, interpretation of results, participated in data discussion and critically revised the manuscript. PC obtained funding, contributed to the study conception and design, provided interpretation of results, and was involved in manuscript writing and editing. PB contributed to the study setup and experimental design, monitored the experimental activity, participated in data discussion, and critically revised the manuscript.

### Conflict of Interest

The authors declare that the research was conducted in the absence of any commercial or financial relationships that could be construed as a potential conflict of interest.
